# Autofluorescence study and selected cyanidin quantification in the Jewel orchids *Anoectochilus* sp. and *Ludisia discolor*

**DOI:** 10.1371/journal.pone.0195642

**Published:** 2018-04-12

**Authors:** Ranjetta Poobathy, Rahmad Zakaria, Vikneswaran Murugaiyah, Sreeramanan Subramaniam

**Affiliations:** 1 School of Biological Sciences, Universiti Sains Malaysia, Gelugor, Pulau Pinang, Malaysia; 2 School of Pharmaceutical Sciences, Universiti Sains Malaysia, Gelugor, Pulau Pinang, Malaysia; Bhabha Atomic Research Centre, INDIA

## Abstract

*Anoectochilus* sp. and *Ludisia discolor* are known as Jewel orchids. Both species are terrestrial wild orchids that grow in shaded areas of forests. The Jewel orchids are renowned for the beauty of their leaves, which are dark-green laced with silvery or golden veins. The orchids are used as a cure in various parts of Asia. Overharvesting and anthropogenic disturbances threaten the existence of the Jewel orchids in the wild, necessitating human intervention in their survival. An understanding of the structure and adaptations of a plant may assist in its survival when propagated outside of its habitat. In this study, *ex vitro* leaves of *Anoectochilus* sp. and *L*. *discolor* were subjected to freehand sectioning, and then inspected through brightfield and fluorescence microscopy. The study indicated that all parts of both plants presented typical monocotyledonous characteristics except the leaves. The leaves displayed dorsiventrality with distinct palisade and spongy mesophyll layers. The spongy mesophyll layer contained cells which fluoresced a bright red when exposed to ultraviolet, blue, and green light wavelengths, hinting at the presence of anthocyanins for photoprotection. Cyanidin was detected in the leaves of *L*. *discolor*, as enumerated through high performance liquid chromatography (HPLC). The observations indicated that *Anoectochilus* sp. and *L*. *discolor* are well-adapted to live under shaded conditions with minimal exposure to light.

## Introduction

The Orchidaceae, composed of about 20,000 orchid species, is the biggest family of flowering plants in the division Magnoliophyta. Members of Orchidaceae are noted for their diversity in terms of the unique adaptations found in their anatomy and morphology [1]. Orchids of subtribe Goodyerinae possess stems that are decumbent or rhizomatous, encircled by a long-lasting rosette of leaves [2]. The Jewel orchids are the most famous members of the group, as seen for *Anoectochilus*, *Dossinia*, *Goodyera*, *Ludisia* and *Macodes* [1]. *Anoectochilus* and *Ludisia* are terrestrial orchids that can be found in various tropical locations in Asia Pacific region [3–7]. The orchids thrive at altitudes ranging from 400–1200 m, under low light conditions, and grow on leaf litters, mossy rocks situated near streams as well as slopes of loamy soil or red clay [8, 9]. The perennial plants grow slowly, with seedlings maturing after two to three years [6, 7, 9].

Several *Anoectochilus* species are used in Chinese folk medicine, such as *A*. *formosanus*, *A*. *koshunensis* and *A*. *roxburghii*. Whole dried or fresh *Anoectochilus* plants are traditionally boiled in water and consumed orally to treat symptoms and diseases such as chest and abdominal pains, diabetes, nephritis, fever, hypertension, impotence, liver and spleen disorder, and pleurodynia [10, 11]. *L*. *discolor* was mixed with *Pholidota chinensis* and administered to patients for eliminating coughs and strengthening weak lungs [12, 13]. The Li ethnic group of Mount Yinggeling, Hainan Island prepares an extract by toasting whole *L*. *discolor* plants to be massaged onto external injuries [14]. The orchids are believed to help reduce aging in the Lao community [7]. *L*. *discolor* is said to be resistant to heat, insects and diseases, contrary to *A*. *formosanus*. Hence, Chou and Chang [13] created a hybrid of the two orchids (*Ludochilus*) to retain the medicinal qualities of *A*. *formosanus*, while expressing the robustness of *L*. *discolor*.

According to Ong and O'Byrne [4], five *Anoectochilus* species are endemic to Peninsular Malaysia: *A*. *falconis* [4, 15], *A*. *reinwardtii*, *A*. *burmannicus*, *A*. *geniculatus* and *A*. *sangineus*. *Anoectochilus* spp. and *L*. *discolor* are valued in horticulture due to their dark coloured and velvety-textured foliage streaked with golden veins [16, 17]. Two *Anoectochilus* species found on Peninsular Malaysia were categorised as data deficient: *A*. *falconis*, last found in 1915 on Gunung Jerai, Kedah; and *A*. *pectinatus*, last found in 1924 from Gunung Hijau, Perak. Data deficient species are at risk of extinction from overharvesting, habitat loss and extreme rarity, hence the necessity in their conservation [4]. Data on the availability of *L*. *discolor* in Malaysia are scarce. In Taiwan, overharvesting of wild *Anoectochilus* spp. plants has reduced its numbers in the wild. Problems associated with the decreasing populations of this orchid include reduced gene flow, inbreeding depression, and reduced fitness [18–20].

The ecophysiological condition of any forest species is largely determined by sunlight intensity. Topmost canopy layers and locations exposed to full sunlight may be exposed to photosynthetically active radiation (PAR) ranging from 1000–2500 μmol m^−2^ s^−1^, while the forest understory may be illuminated with PAR below 5 μmol m^−2^ s^−1^ [21]. “Shade” plants may present in the form of a “true” or obligate shade plant: entire species or groups of plants that are not able to tolerate high light conditions; a “shade-adapted” plant: certain genotypes of a plant species that have adapted to shade conditions; and a “shade form” of a plant: plants within a particular species that possess a phenotype corresponding to growth under shady conditions. Shade plants may therefore possess an “adapted” or an “acclimated” phenotype, both collectively termed as facultative. The term “shade leaf” hence refers to leaves grown under low light intensities [22].

Light intensity plays a major role in determining the leaf structure of a species. The arrangement and shapes of the chlorenchyma cells vary in different plant species [21]. In the case of obligate shade plants, specific anatomical adaptations assist in enhancing light absorption [22]. Light can hence be a negative or a positive influence, the former in the form of stress factors resulting from too much or too little light. Excessive radiation results in the over-energisation of the photosynthetic apparatus, causing photoinhibition and/or photodestruction, while inadequate illumination causes limitations in the production of energy from photosynthesis [23]. Anthocyanins, typically found in the leaves of some plant species, were said to be able to scavenge free radicals generated through environmental stressors such as spikes in UV radiation levels or saturating light fluxes [24, 25], and by absorbing a fragment of the light illuminating the foliage [26].

Anthocyanins, a subset of flavonoid-derived compounds, are water-soluble plant flavonoids that bestow colours ranging from scarlet to blue in flowers, fruits, leave and plant storage organs [27, 28]. Anthocyanin compositions in wild plants conform to a mutation pattern that begins with delphinidin, followed by cyanidin and then pelargonidin, with the former found in plants that evolved earlier. Delphinidin, cyanidin and their derivatives are known to display a violet colour in many plant organs [29]. Studies in elucidating the roles of anthocyanic compounds in the leaves of the orchids are scarce [30, 31]. The role of anthocyanins in the case of *Anoectochilus* spp. and *Ludisia discolor* remains a mystery.

This study aims to visualise the ultrastructure of *Anoectochilus* sp. and *L*. *discolor* through freehand sectioning and autofluorescence observations in order to understand the structural and environmental adaptations of both the Jewel orchids. This study also aims to quantify the amount of cyanidin present within the leaves of *L*. *discolor* as a confirmation of the presence of anthocyanins within the leaves of the Jewel orchids. It is hoped that this study is able to contribute towards efforts in mitigating the dwindling numbers of *Anoectochilus* sp. and *L*. *discolor* in Peninsular Malaysia, allowing the orchids to thrive under human intervention.

## Materials and methods

### Plant sourcing for wild *Anoectochilus* sp. and *L*. *discolor*

Wild *Anoectochilus* sp. plants were collected from Penang Hill at an elevation of 600–750 m. Sampling was approved by the Penang State Forestry Department, Malaysia, and conducted every two months from March 2012 until April 2015. The orchid plants, estimated at two to three years of age, were found amidst leaf litter located in the forest understory. Fully-formed inflorescences were not observed throughout the study, making the conclusive identification of the specific epithet of the *Anoectochilus* sp. difficult. Harvested *Anoectochilus* sp. plants were stored in a white plastic container filled with soil collected from the sample site and wetted with tap water. Collected plants were stored under cool white fluorescent lamps (Philips TLD, 36 W, 150 μmol m^-2^ s^-1^), shaded to reach a photon flux density (PFD) of 3.4 μmol m^-2^ s^-1^, and following 16 hours photoperiod until subsequent repotting attempts. Wild and mature *L*. *discolor* plants, estimated at two to three years of age, were gifted by Dr. Rahmad Zakaria, who sourced the plants from Sungai Enam, Temenggor, Perak, Malaysia. Both the orchid plants were identified by Dr. Rahmad Zakaria of the School of Biological Sciences, Universiti Sains Malaysia and Mr. Ong Poh Teck of the Forest Research Institute Malaysia (FRIM). Voucher specimen of both *Anoectochilus* sp. and *L*. *discolor* were deposited at the herbarium of Universiti Sains Malaysia and given the accession numbers L/C 005 and L/C 006 respectively.

**Fig 1:**
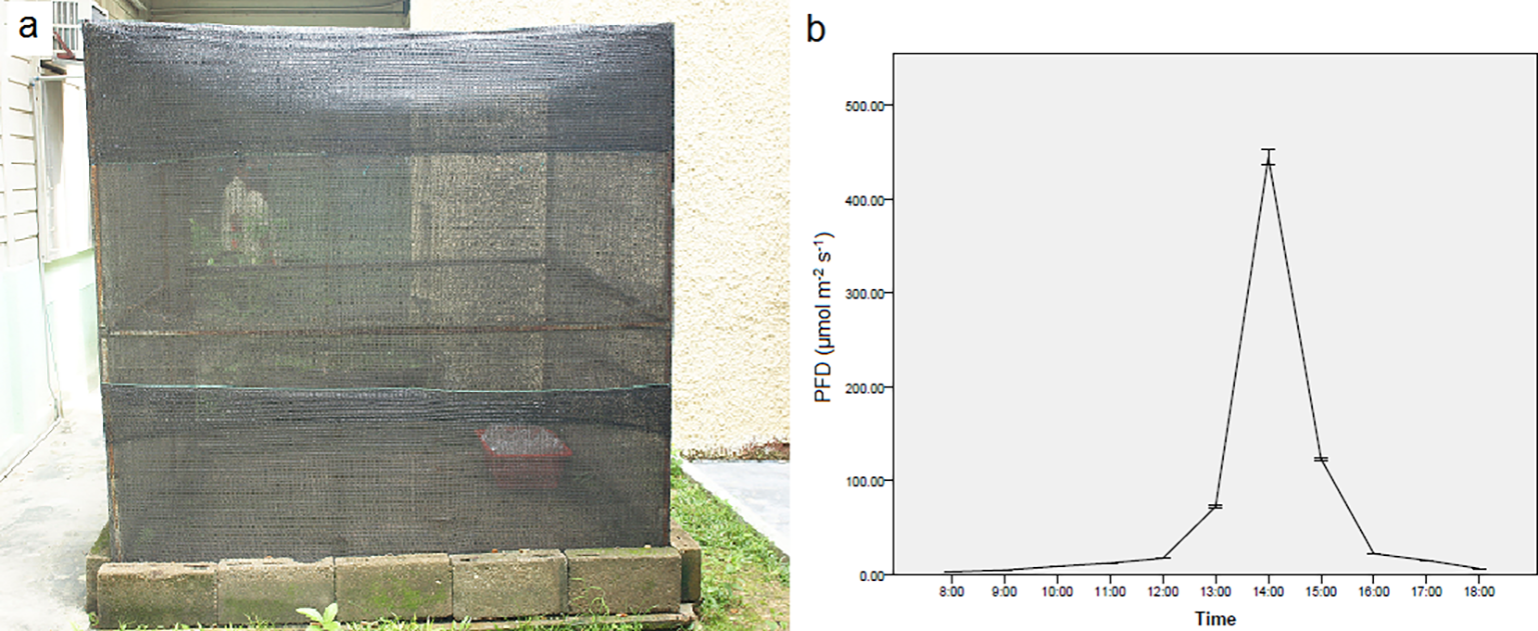
Shade house conditions for the Jewel orchids *Anoectochilus* sp. and *L*. *discolor*. **(a)** The shade house was covered with layers of shade nets that reduced sunlight penetration down to 22.4%. **(b)** Photon flux density (PFD) recorded within the shade house every daylight hour.

Both orchid plants were acclimatised under shade house conditions in perforated plastic pots containing peat moss, coconut coir and coconut husks (1:1:1) at the School of Biological Sciences, Universiti Sains Malaysia. The protocol was adapted from Shiau *et al*. [7] and Ket *et al*. [32]. The 2 m × 2 m shade house (Fig 1A) was covered with overlapping layers of shade nets that reduced sunlight penetration to 22.4%, in order to mimic the conditions of the sampling location at Penang Hill. The lowest and highest PFD within the shade house, following a 13/11 natural photoperiod, was recorded at (mean ± standard deviation) 2.65 ± 0.02 and 444.62 ± 13.23 μmol m^-2^ s^-1^ respectively, corresponding to sunrise at 8 am and the afternoon sun at 2 pm local time (Fig 1B; Extech EA30: EasyView™ Wide Range Light Meter, Extech Instruments). The relative humidity and temperature of the shade house were allowed to fluctuate as per the local conditions. The plants were watered three times a week with tap water. Freehand sections used in this study were obtained from plants that were successfully acclimatised for a year.

### Sample selection

**Fig 2:**
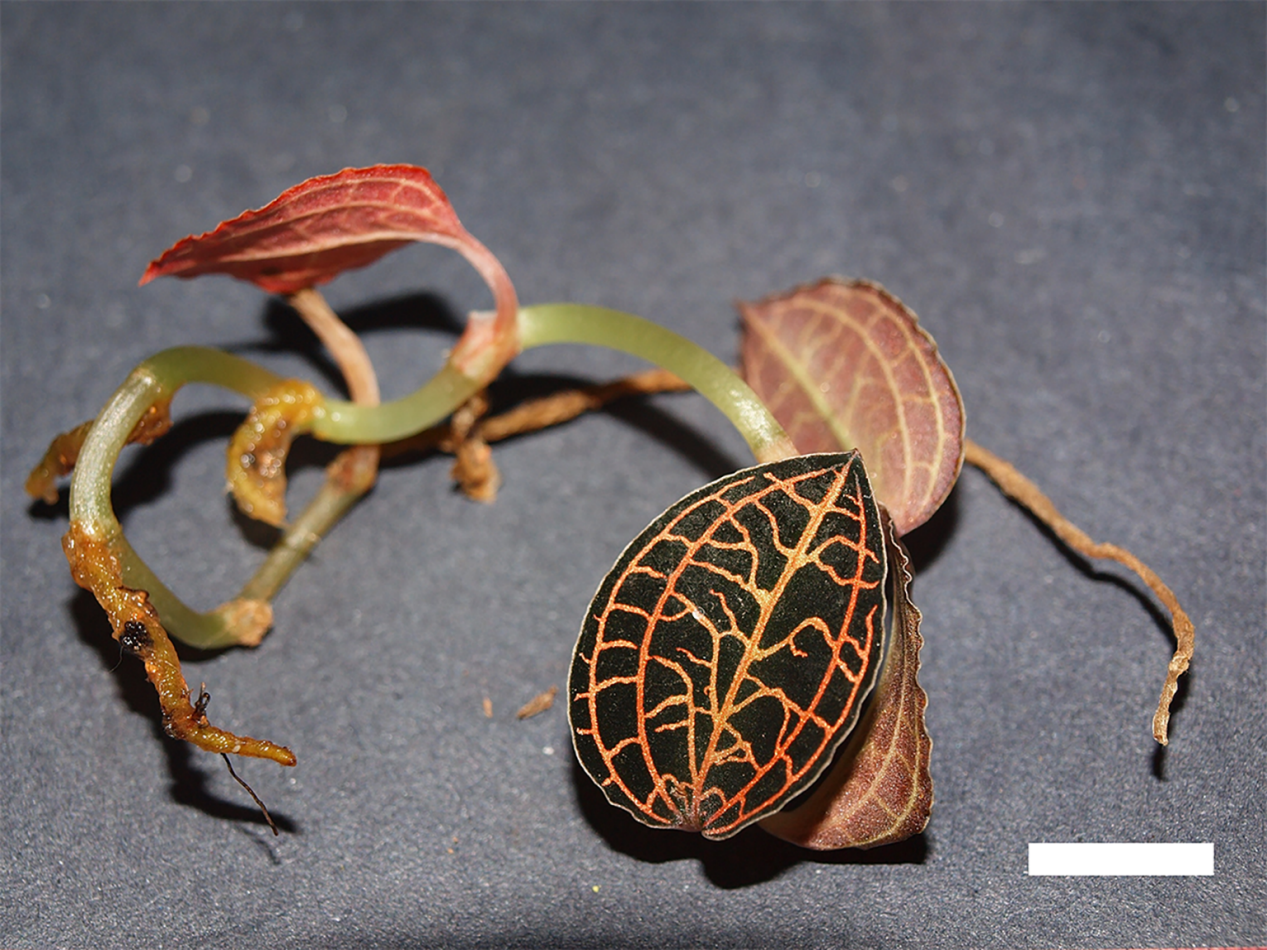
A mature *Anoectochilus* sp. plant. Leaves of the plant displayed a dark green-black adaxial surface laced with golden veins, and a red abaxial layer. Bar = 1 cm.

**Fig 3:**
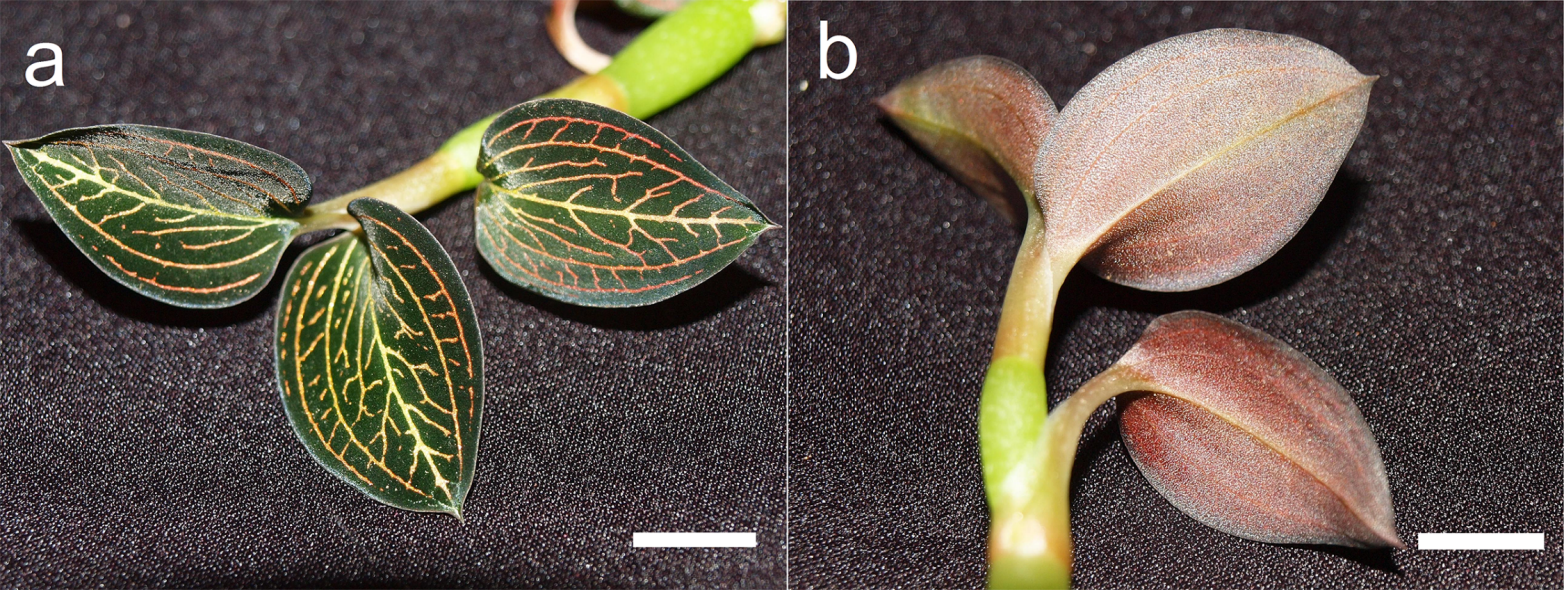
A mature *L*. *discolor* plant. Leaves of the plant displayed a dark green adaxial surface laced with golden veins, and a red abaxial layer. Bar = 1 cm.

Samples were obtained from *ex vitro* leaves of *Anoectochilus* sp. (Fig 2) and *L*. *discolor* (Fig 3). Leaves were preferentially selected from both the plants, in which only fully-expanded leaf laminae were chosen for the observation [33]. Cross sections of all samples were made in the middle region of the target tissues. The observations were conducted eight times over a period of 14 months, from January 2015 to February 2016. In the case of *L*. *discolor*, three leaves were selected per plant and three plants were selected for each observation session. A minimum of two leaves were selected from a minimum of two plants for observations involving *Anoectochilus* sp., due to sample scarcity. Leaf collection was conducted at noon. All chemicals used in this study were sourced from R&M Chemicals, Essex, UK, unless stated otherwise.

### Freehand sectioning

The freehand sectioning technique was adopted from Yeung [34]. Double-edged razor blades used for sectioning were flooded with distilled water prior to slicing the samples. To obtain sections of both abaxial and adaxial regions of the leaves, the leaves were torn at an oblique angle. The thinnest sections were selected for observation [34].

### Autofluorescence characterisation of leaves

Transverse and longitudinal hand sections were prepared from the leaves of both *Anoectochilus* sp. and *L*. *discolor*. The sliced segments were mounted in 10% sucrose, covered with a cover slip and then viewed using bright field microscopy under 40×, 100×, 200×, 400× and 1000× magnifications. The samples were viewed under brightfield illumination prior to shifting to epifluorescence to locate coloured components of the leaf cells. The distribution of red pigmentation found in various locations of the leaves was assumed to correspond to the presence of anthocyanin [35]. To test the hypothesis that some cells may contain anthocyanin that is subdued by local variation in the vacuolar pH, the sliced segments were infiltrated in 1 M HCl following mounting in 10% sucrose [35, 36].

### Imaging and photography

Leaf sections of *L*. *discolor* and *Anoectochilus* sp. were captured using a fluorescence microscope (Olympus BX51, Olympus Corporation, Tokyo, Japan) fitted with the UIS2 optical system composed of broad range ultraviolet (UVW), blue (BW), and green (GW) excitation lenses and affixed to a DP72 colour video camera (Olympus Corporation, Tokyo, Japan). Image analyses were performed using cellSens imaging software (Olympus Corporation, Tokyo, Japan).

### Sample preparation for high performance liquid chromatography (HPLC)

*Ex vitro* plants of *L*. *discolor* were selected for this study. *Anoectochilus* sp. was exempted from this study due to the lack of explants. Fresh *ex vitro* leaves of *L*. *discolor* were dissected with a pair of scissors and briefly rinsed to remove debris. The leaves were dried with kitchen towels and then weighed (Cubis^®^ Precision Balance, Sartorius AG, Göttingen, Germany). The leaves were dried for one week in the dark at 40°C (Memmert UFE 400, GmBH + Co. KG, Germany). The dried leaves were then stored in a desiccator filled with silica gel for a week. Dry weight measurements were conducted thrice. The leaves were then pulverised in a domestic electric blender (Panasonic MX-900M, Panasonic, Malaysia) to a powdery consistency. The powdered sample was stored in 50 mL centrifuge tubes (BD Falcon™, Corning, New York, USA) and placed in a container filled with silica gel until the extraction step.

The powdered explant sample was weighed (1 g) and placed in pre-cleaned and pre-weighed (M-220, Denver Instrument) boiling tube, which was filled with 10 mL 100% methanol, following a sample to solvent ratio of 1:10 (w/v). The boiling tube was sealed with aluminium foil and rubber bands, and placed in a sonicator (Citizon Ultrasonic cleaner, Model YJ5120, 3200DT, Citizon Scale 1 Pvt. Ltd., Mumbai, India), set at room temperature, for 20 minutes. The sample was then allowed to stand for 15 minutes. The mixture was filtered into a pre-cleaned sample vial (21 mm × 85 mm) using a piece of filter paper (Whatman^®^ Grade 1 Qualitative Filtration Papers, Whatman, Inc., New Jersey, USA). The filtrate was placed in the oven (Memmert UFE 400, GmBH + Co. KG, Germany) set at 45°C for drying. The entire process was repeated twice. All filtrates were collected and stored in a vial, and allowed to evaporate in the oven for four days, until the solvent completely dried off. The vial containing the dried extract was weighed, and the weight of the extract was recorded by subtracting the weight of the empty vial from that of the combined weights of the vial and the sample. A minimum of 10 mg of the extract was removed from the sample vial and placed into a 2 mL microcentrifuge tube, and was resuspended to a concentration of 10 mg mL^-1^ with 100% methanol acidified with HCl (0.1% v/v).

The formula below was used in calculating dry yields from fresh samples:

$$Percentage\;yield\;(\%)=\frac{x_{1}}{x_{0}}\times 100$$

Where *X*_1_ = mass of dry sample (g)

                *X*_0_ = fresh weight of sample (g)

The following details were used when the extraction yields were calculated:

                *X*_1_ = mass of dry methanolic extract (g)

                *X*_0_ = mass of dry sample (g)

### HPLC analysis on leaf extract of *L*. *discolor*

The protocol used in this study was modified from Jing *et al*. [37]. The methanolic leaf extract of *L*. *discolor* were analysed and monitored at 280nm using a high performance liquid chromatography (HPLC) system on an e2695 Separations Module (Waters, Massachusettes, USA) equipped with an autosampler, a 2489 UV/Visible Detector, and the Empower software installed on a Lenovo ThinkCentre (North Carolina, USA) workstation. A 5 μM Zorbax Eclipse Plus Phenyl-Hexyl column (4.6×250 mm, Agilent Technologies, California, USA) pre-fitted with a 4.6×12.5 mm Zorbax Eclipse Plus-C18 5 μM analytical guard column (Agilent Technologies, California, USA) was used as the stationary phase. The mobile phase used in the analysis consisted of 0.5% acetic acid (R&M; Essex, UK) as solvent A and 100% methanol (HPLC grade, Fisher Chemical, Massachusettes, USA) as solvent B. The solvents were filtered with either HA-grade 0.45 μm hydrophobic polytetrafluoroethylene (PTFE) or FH-grade 0.45 μm mixed cellulose ester membranes (Millipore; Merck, Darmstadt, Germany). The samples were prepared at 5000 ppm in acidified methanol (0.1% HCl, v/v) and were filtered through a 0.45 μM polypropylene filter (Whatman, Inc., New Jersey, USA). The samples were separated using an isocratic method (A:B = 60:40) utilising a 1 mL min^-1^ flow rate and a 10 μL injection volume. Cyanidin chloride (ChemFaces, Wuhan, China) was assigned as the standard and suspended in acidified methanol (0.1% HCl, v/v) at 1000 ppm, and the maximum absorption spectrum of the pure compound was determined over a wavelength range of 200–600 nm (Lambda 25 UV/Vis Spectrometer; PerkinElmer, Massachusettes, USA). All analyses were conducted in triplicates. Data were enumerated using mean and standard deviation.

## Results

### Autofluorescence characterisation of leaves

The leaf segments of both orchid plants were viewed immediately after the sectioning procedure. Freehand sections of both *Anoectochilus* sp. and *L*. *discolor* were observed for autofluorescence under broad-spectrum ultraviolet (UVW), blue (BW) and green (GW) wavelengths. Preliminary observations of freehand sections of both wild orchid species indicated that anthocyanin localisation did not occur in all leaf cells. To rule out the possibility that the colour of the anthocyanins was repressed as a result of intercellular pH variation, all segments were immersed in 1 M HCl prior to microscopy observation. No colour changes were observed in cells that were originally colourless.

**Fig 4:**
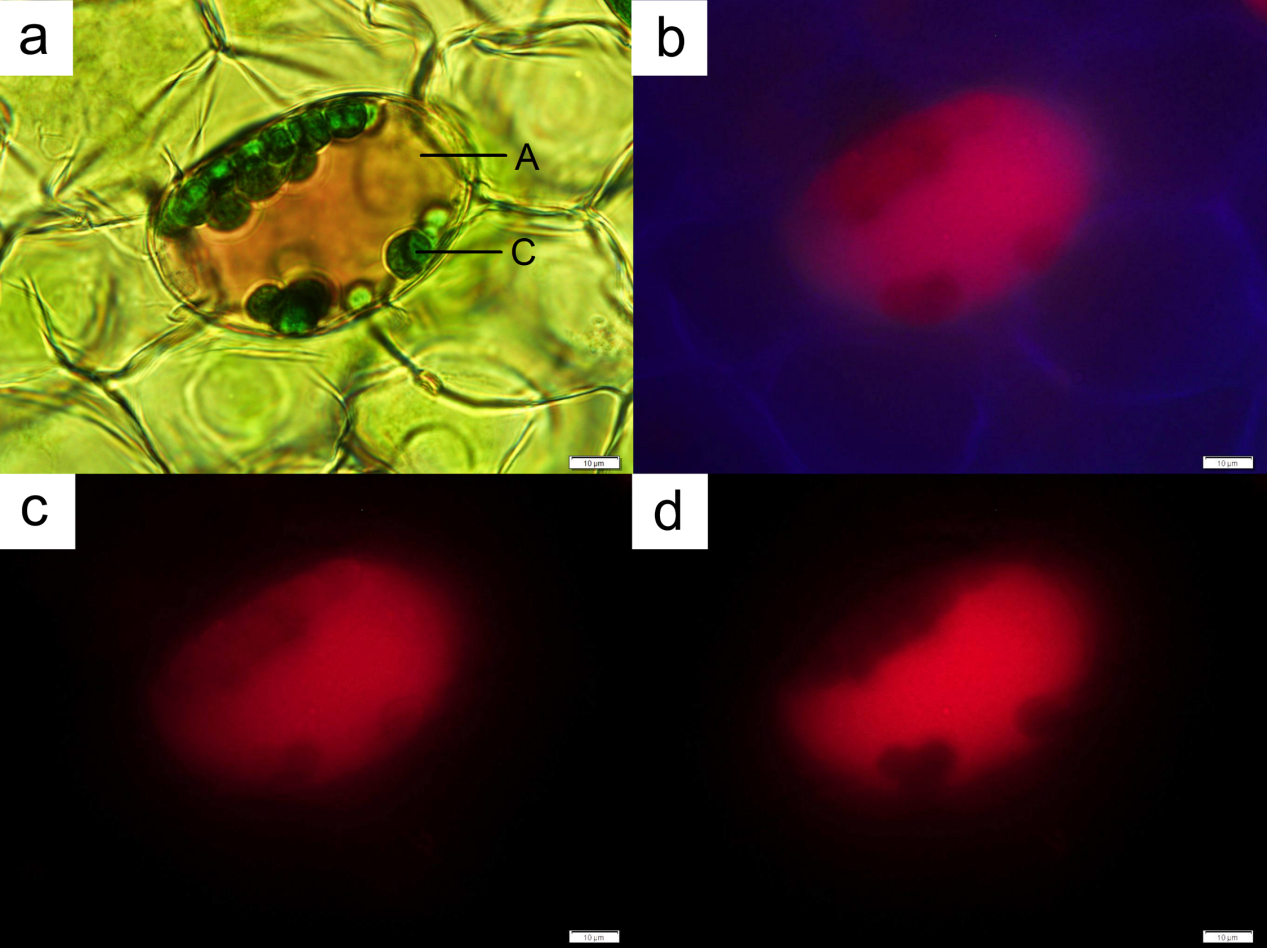
A leaf cell from *Anoectochilus* sp. The cell contained both chloroplasts (C) and anthocyanins (A; **a**). The contents of the cell fluoresced under both UV ray (**b**) and blue light (**c**). The strongest glow from the anthocyanins was obtained under green light (**d**). Bar = 10 μm.

**Fig 5:**
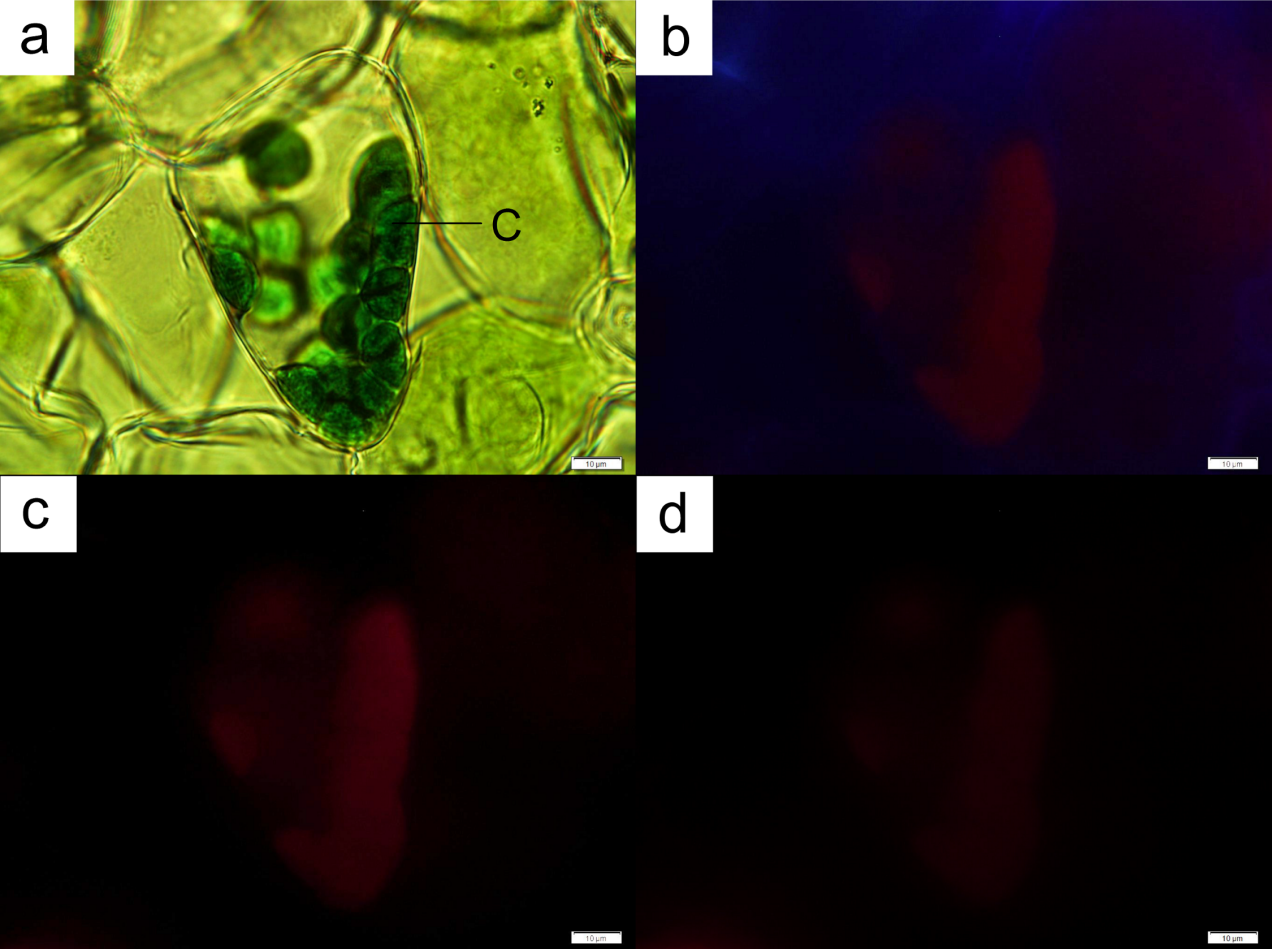
A leaf cell from *Anoectochilus* sp. The cell contained only chloroplasts (C; **a**). The chloroplasts fluoresced under both UV ray (**b**) and blue light (**c**), with the strongest glow obtained from the latter. The weakest glow was obtained under green light (**d**). Bar = 10 μm.

Both orchid species presented a prominent purple-red abaxial leaf layer (Figs 2 and 3). Anthocyanins were observed to be contained in vacuoles that occupy the entire cellular space in both orchid species (Fig 4A). Chloroplasts were found in both anthocyanin-containing cells (Fig 4A), and in cells devoid of the pigment (Fig 5A). The chloroplasts in both types of cells were found to fluoresce strongly under UV (Figs 4B and 5B) and blue light illuminations (Figs 4C and 5C), but weakly under green light (Figs 4D and 5D). However, cells with anthocyanins fluoresced a strong red under green light (Fig 4D), while presenting a muted glow under UV and blue light illuminations (Fig 4B and 4C).

**Fig 6:**
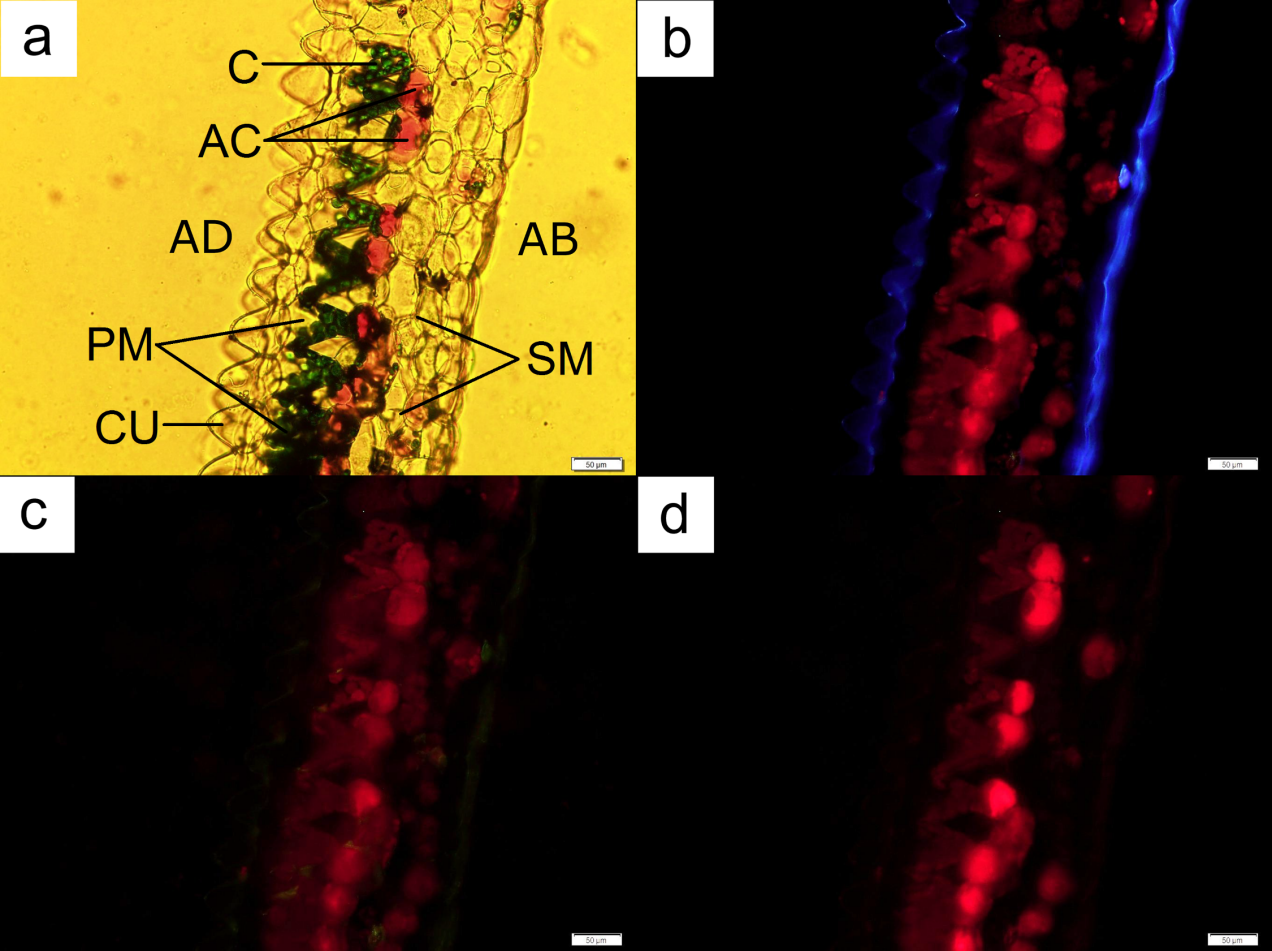
A leaf cross-section from *Anoectochilus* sp. Each palisade mesophyll cell was accompanied by an anthocyanin-containing spherical cell (AC) located directly underneath the former (**a**). The chloroplasts (C) fluoresced under both UV ray (**b**) and blue light (**c**), with the strongest glow obtained from the latter. Anthocyanin-containing cells (AC) fluoresced the strongest under green light (**d**). AB = abaxial; AD = adaxial; CU = cuticle, SM = spongy mesophyll. Bar = 50 μm.

**Fig 7:**
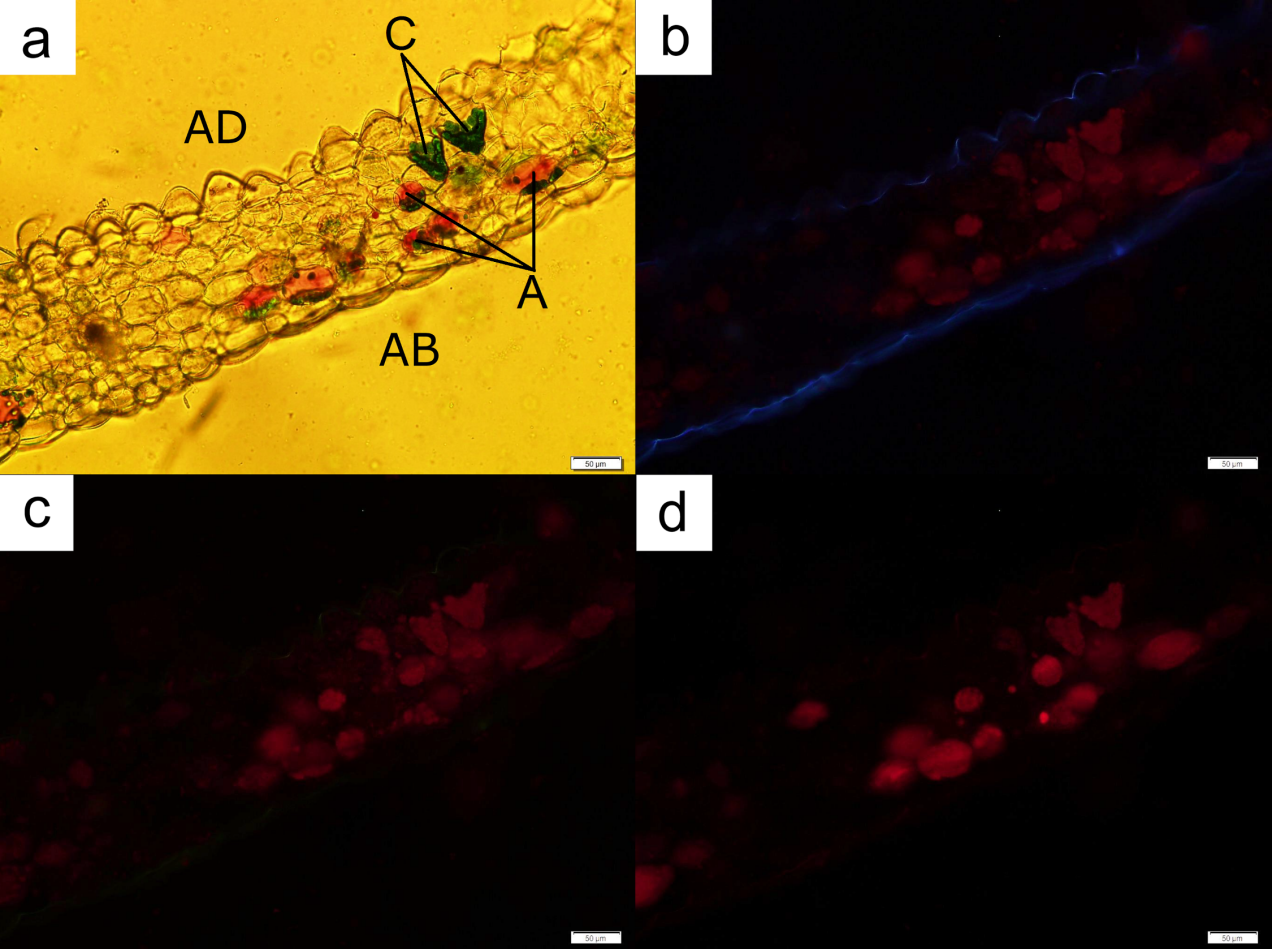
A leaf cross-section from *Anoectochilus* sp. showing the tapering of the palisade layer into a vascular bundle area. The number of cells containing chloroplasts (C) lessened (**a**). The UV rays (**b**) and blue light (**c**) indentified cells containing chloroplasts (C), while green light (**d**) identified cells containing anthocyanins (A). AD = adaxial; AB = abaxial. Bar = 50 μm.

**Fig 8:**
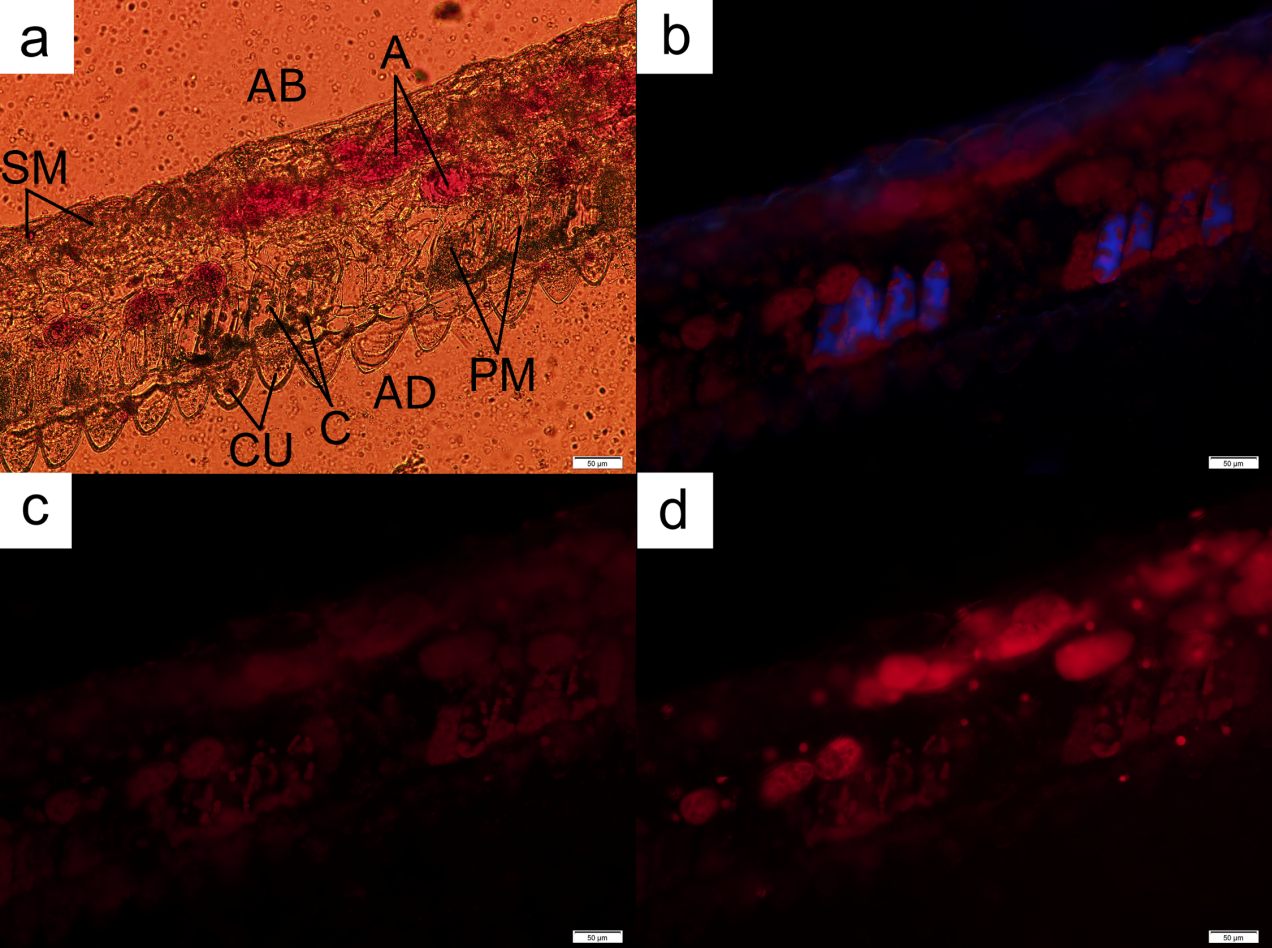
A leaf cross-section from *L*. *discolor*. The palisade mesophyll layer was accompanied, albeit inconsistently, by anthocyanic layers of cells (A) located directly underneath the former (**a**). The chloroplasts (C) fluoresced under both UV ray (**b**) and blue light (**c**), with the strongest glow obtained from the latter. Anthocyanin-containing cells fluoresced the strongest under green light (**d**). AB = abaxial; AD = adaxial; CU = cuticle; PM = palisade mesophyll; SM = spongy mesophyll. Bar = 50 μm.

Anthocyanin localisation in both orchid species occurred in the middle or the abaxial part of the leaves (Fig 6), with the exception of leaf regions possessing the vascular bundle (Fig 7). Cells with anthocyanins were not located at the adaxial layer of the leaf otherwise. Freehand sections of leaves of both orchids again indicated that a distinct cylindrical-shaped chloroplast-dense cell layer, similar to the palisade mesophyll layer in dicotyledonous leaves, was present beneath the cuticle layer of the adaxial region of the leaves (Figs 6 and 8). This layer was followed immediately by a layer of spherical cells containing anthocyanins. Some regions of the observed leaves possessed a single layer of the anthocyanin-containing cells, while other samples displayed multiple layers of such cells.

In the case of *Anoectochilus* sp., each palisade mesophyll cell was accompanied by an anthocyanin-containing spherical cell located directly underneath the former (Fig 6). The palisade mesophyll cells were triangle in shape, with the chloroplasts pooled along the legs of the triangle leading to the vertex pointing towards the abaxial part of the leaf (Fig 6A). The anthocyanin-containing companion cells fluoresced the strongest under green light (Fig 6D) when compared to similar cells located in other sections of the leaf. The base of each cell was connected to a single cuticle at the adaxial part of the leaf, forming a cuticle-palisade cell pair and layer. The abaxial part of the leaf was covered with a layer of clear epidermal cells.

Cross sections of leaves of *L*. *discolor* indicated that the palisade mesophyll cell layer was accompanied by multiple layers of anthocyanin-containing spherical cells located directly underneath the former (Fig 8). However, the arrangements of these cells were not continuous. These anthocyanic cells also fluoresced strongly under green light (Fig 8D) when compared to similar cells located in other sections of the leaf. The chloroplasts in the rectangle-shaped palisade mesophyll cells were situated along the perimeter of the plasma membrane. The base of each cell was connected to a single cuticle at the adaxial part of the leaf, forming a cuticle-palisade cell pair and layer, as observed in *Anoectochilus* sp. The abaxial part of the leaf was covered with a clear epidermal layer.

**Fig 9:**
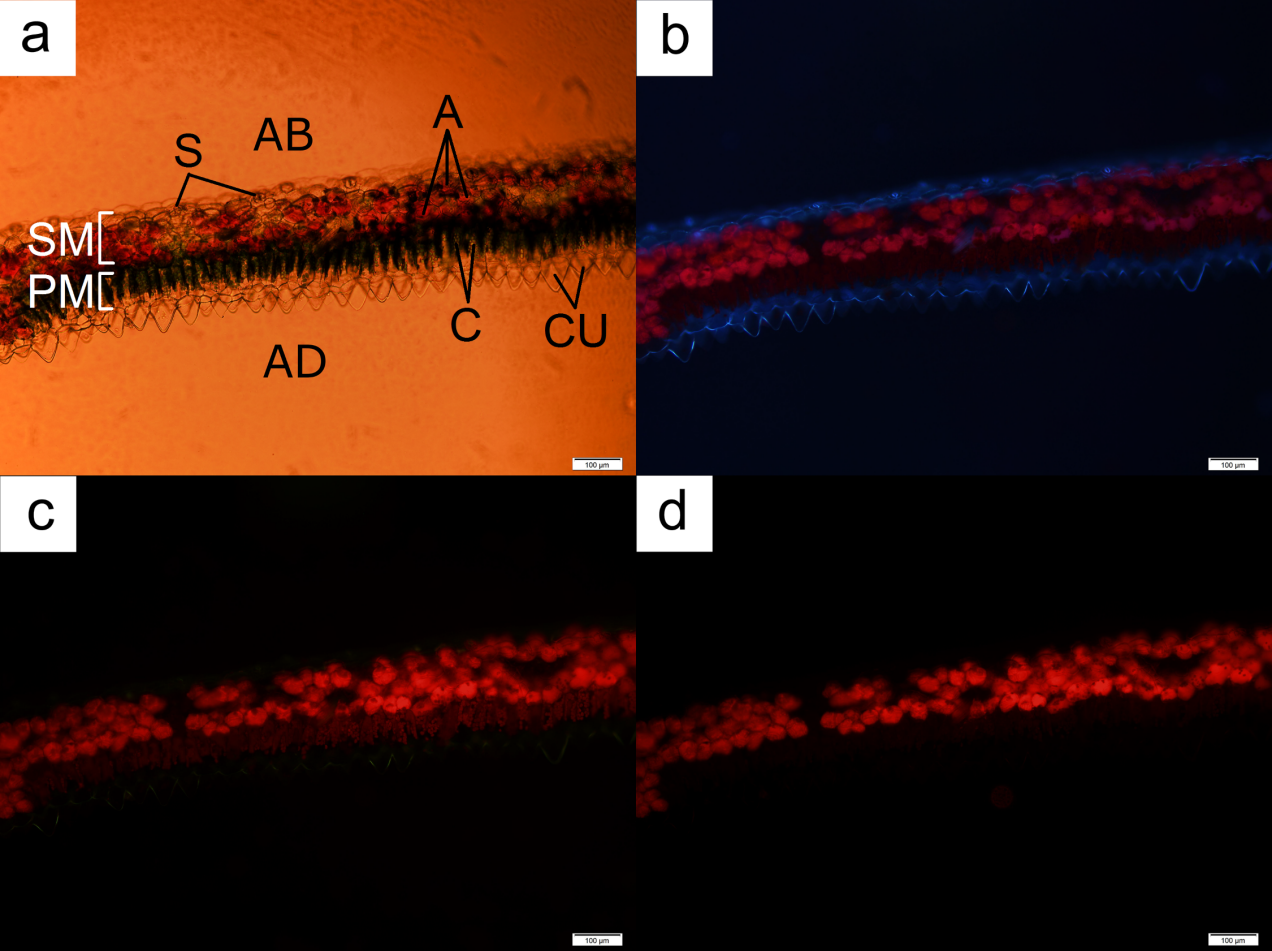
A leaf cross-section from *L*. *discolor* displaying cells with multiple anthocyanic layers. The arrangement of cells containing chloroplasts (C) and anthocyanins (A) were observed under brightfield microscopy (**a**), UV rays (**b**), blue light (**c**) and green light (**d**). AB = abaxial; AD = adaxial; CU = cuticle; PM = palisade mesophyll; S = stoma; SM = spongy mesophyll. Bar = 100 μm.

**Fig 10:**
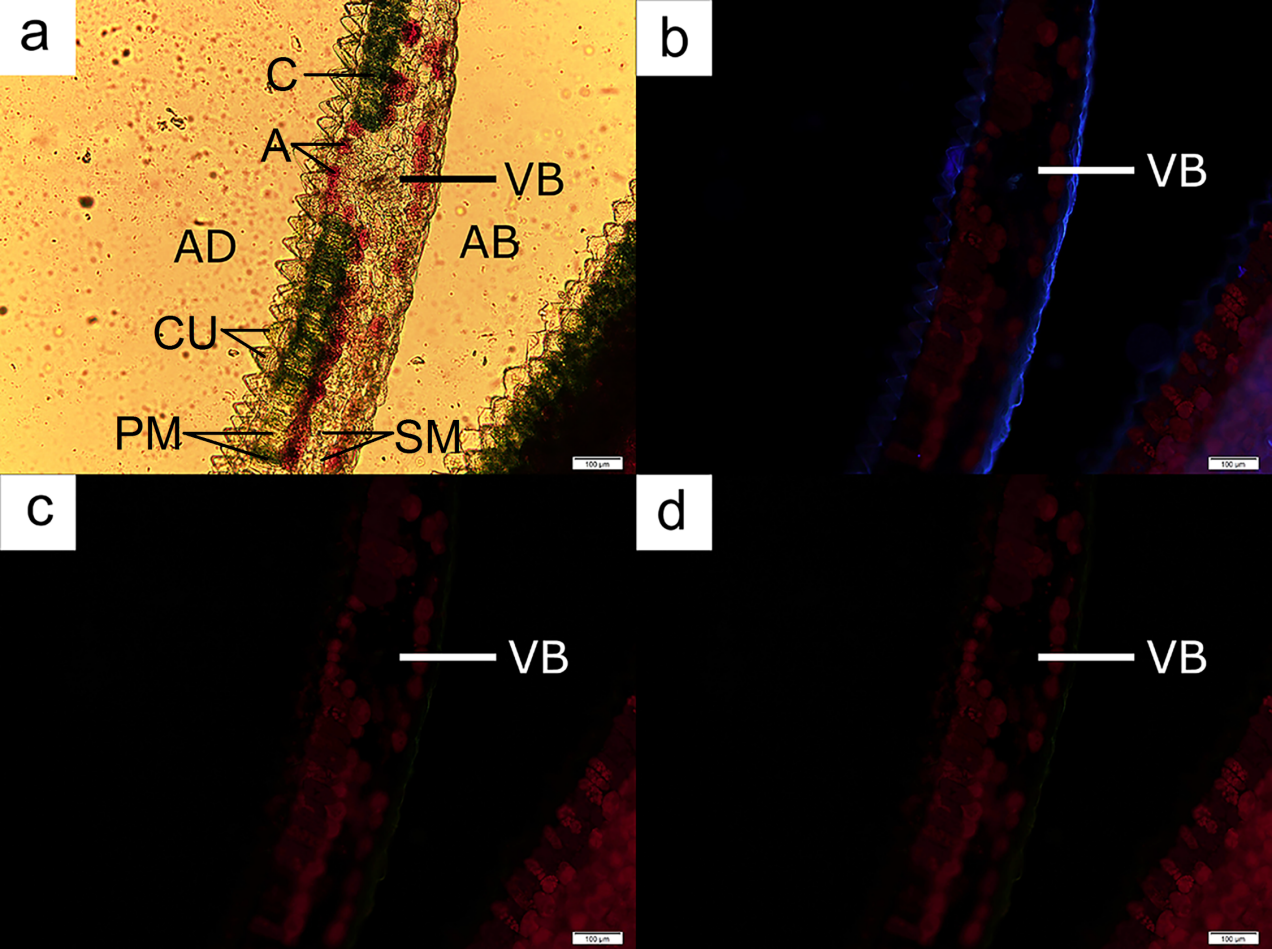
A leaf cross-section from *L*. *discolor* displaying deviation in the anthocyanic layer when vascular bundles are present. The arrangement of cells containing chloroplasts (C) and anthocyanins (A) were conducted under brightfield microscopy (**a**), UV rays (**b**), blue light (**c**) and green light (**d**). AB = abaxial; AD = adaxial; CU = cuticle; PM = palisade mesophyll; SM = spongy mesophyll; VB = vascular bundle. Bar = 100 μm.

The leaves of *L*. *discolor* displayed multiple anthocyanic arrangements. Some sections displayed two distinct anthocyanic layers (Fig 8), while others displayed multiple rows of cells containing anthocyanins (Fig 9). Under all circumstances, the anthocyanic cells were always found beneath the palisade layer, with the exception of regions containing the vascular bundle, in which anthocyanic were located at the adaxial part of the leaf (Fig 10).

### High performance liquid chromatography (HPLC) analysis on the leaf extract of *L*. *discolor*

A total of 65.374 g fresh leaves were harvested from 54 *ex vitro L*. *discolor* plants. Leaf production ranged from 3–7 blades per plant. The leaves weighed 5.963 g after the oven-drying treatment, with the water content of the leaves recorded at 90.9%. The yield obtained from the methanolic extraction of the dried powdered leaves was 0.286 g.

**Fig 11:**
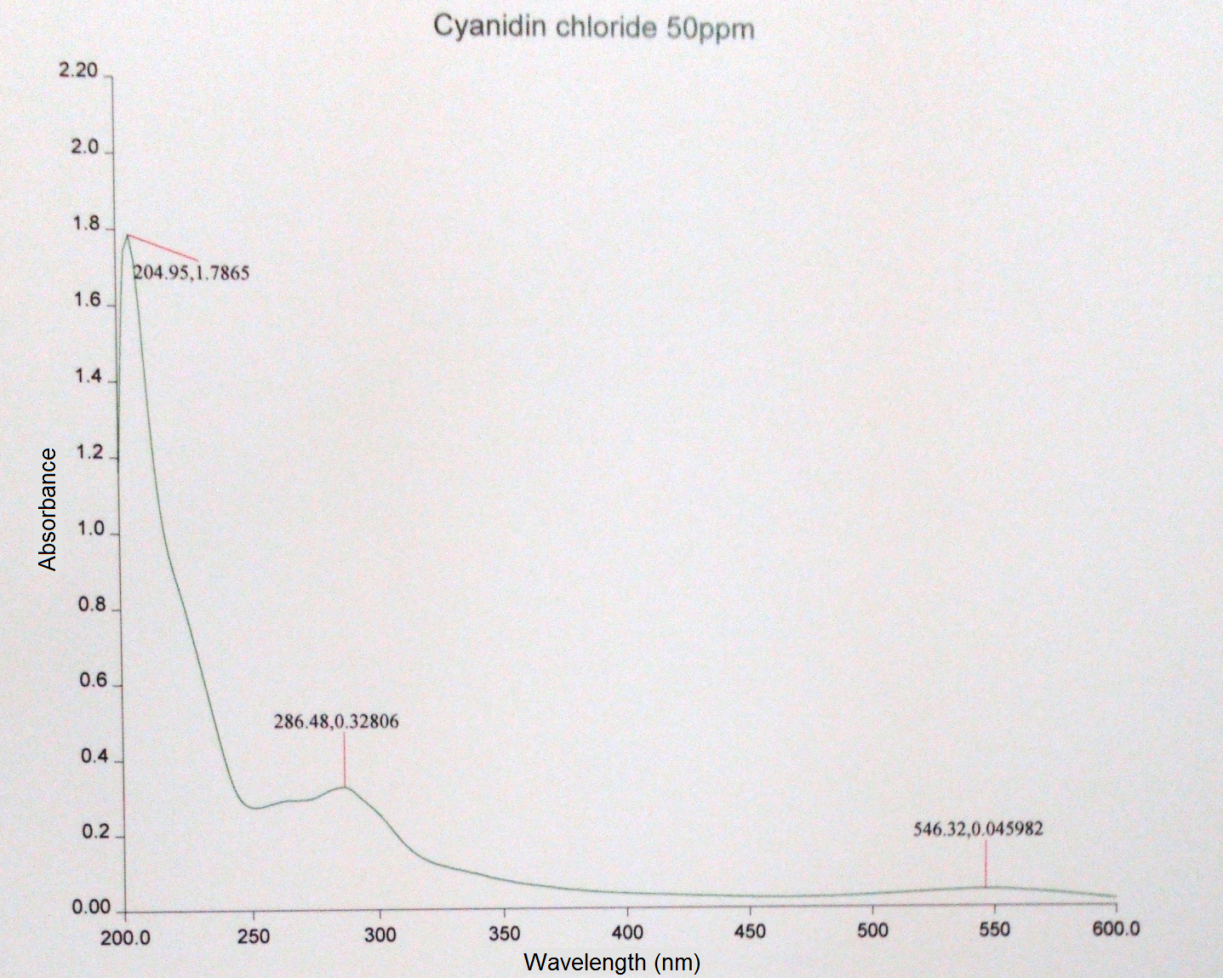
UV-visible spectrum of cyanidin chloride at 50 μg mL^-1^.

**Fig 12:**
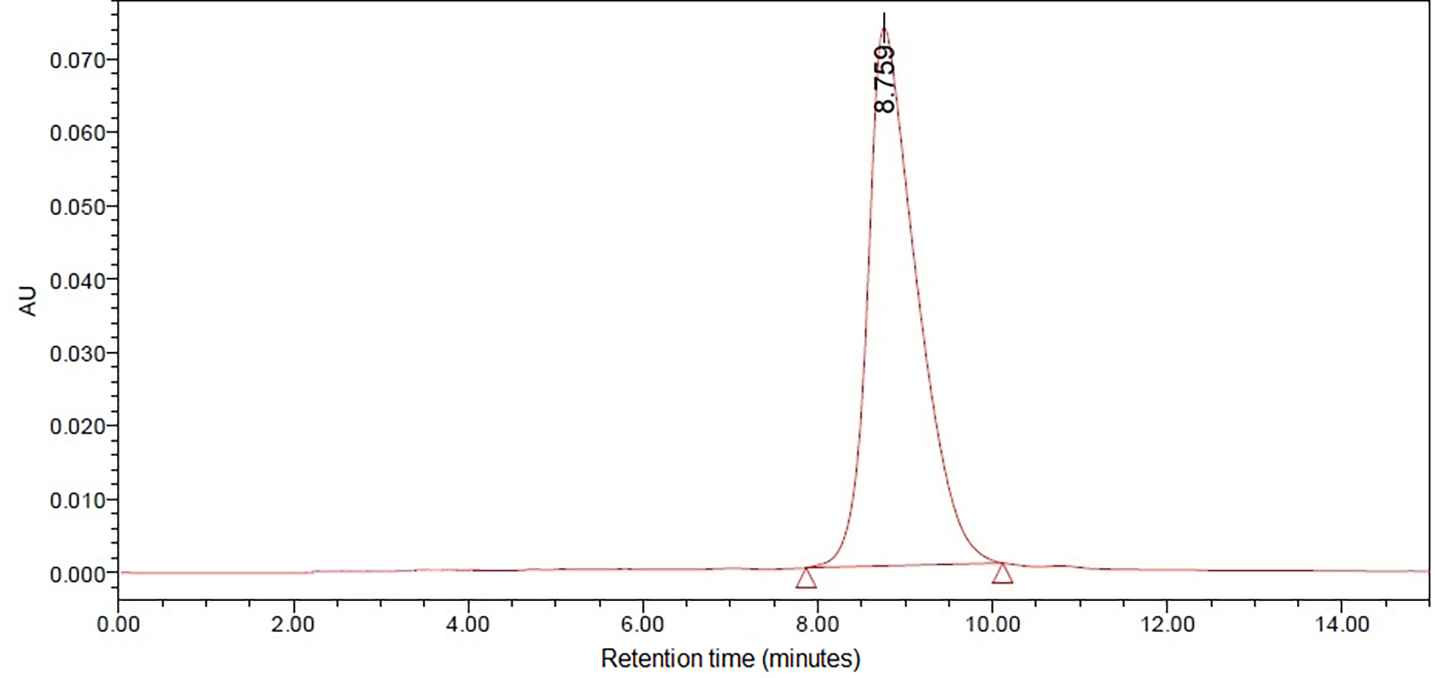
HPLC chromatogram of the cyanidin chloride standard at 1 mg mL^-1^.

**Fig 13:**
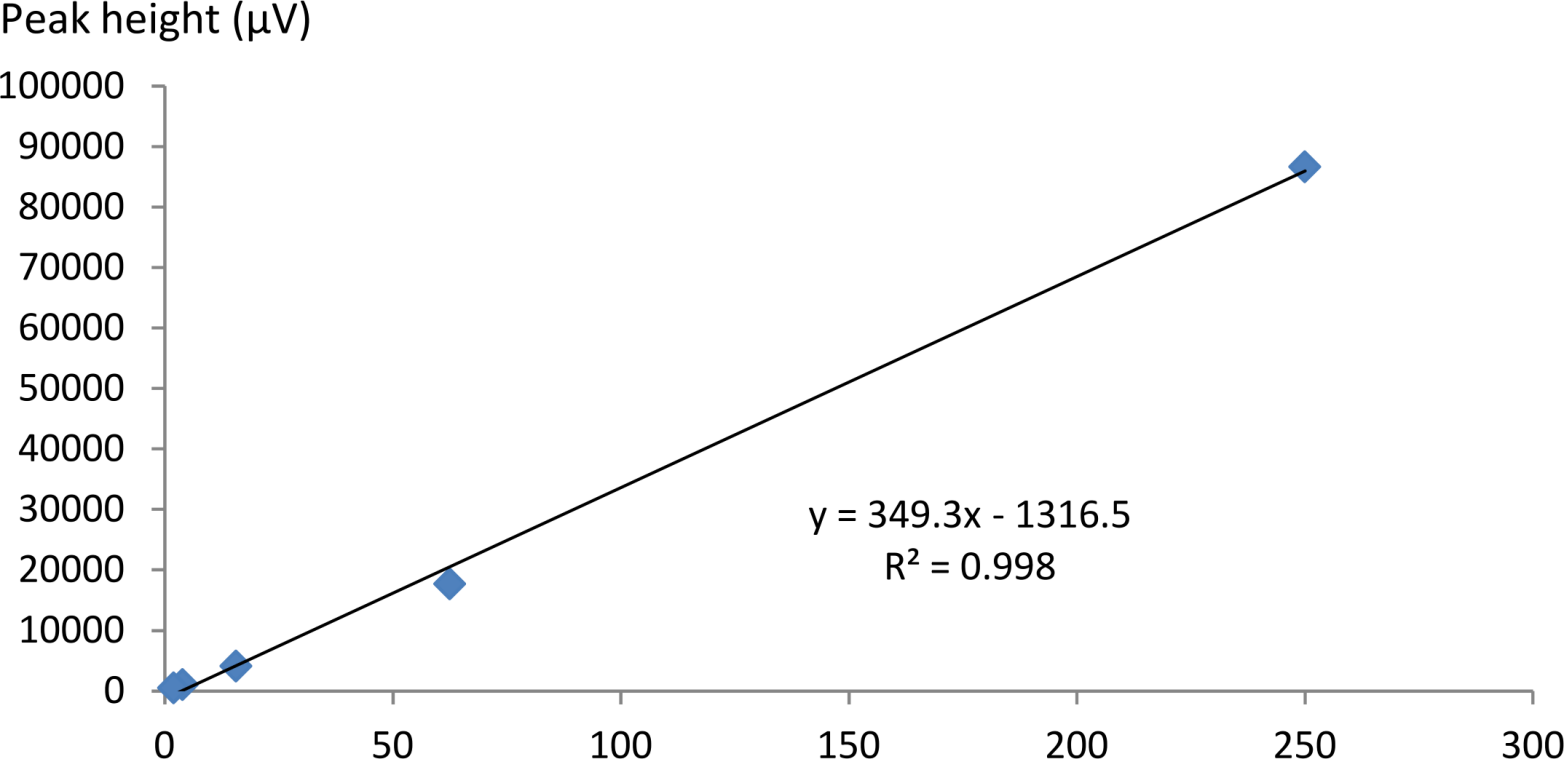
Calibration curve of the cyanidin chloride standard.

The UV visible spectrum of the cyanidin chloride standard indicated that the compound possessed two absorption maxima: one at 286.48 nm (absorption = 0.32806) and another at 546.32 nm (absorption = 0.045982; Fig 11). The former was selected and modified to 280 nm for the experiment due to the higher absorption value. The target peak in each sample was detected based on the congruence of the retention times between the standard peak (Fig 12) and the corresponding peak in the methanolic extract. Enumeration of cyanidin chloride in the sample was conducted through an equation generated from the calibration curve of cyanidin chloride detected at 280 nm (Fig 13).

**Fig 14:**
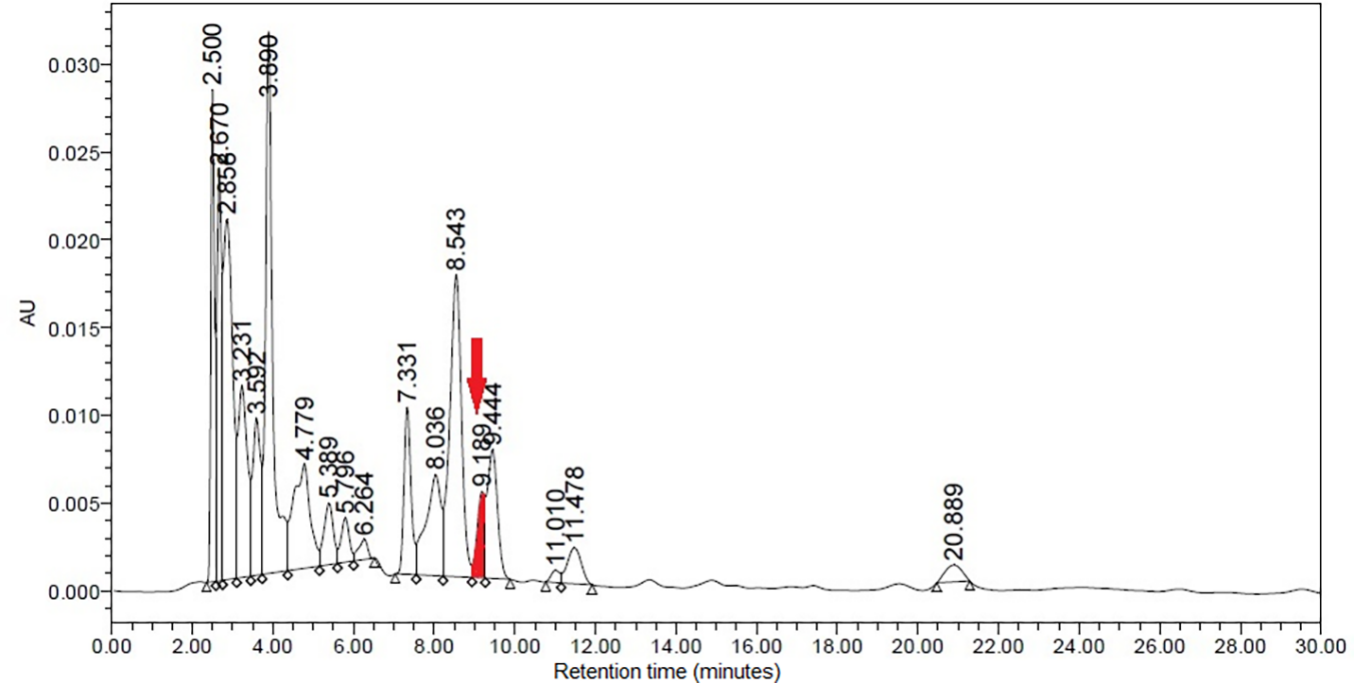
Chromatogram of the methanolic extract of *L*. *discolor* at 5 mg mL^-1^. The target peak is shaded and marked with an arrow.

The majority of the peaks eluted out before the 13-minute mark when the methanolic extract was subjected to HPLC, with the final peak eluting out before the 22-minute mark. Peaks that eluted before the 10-minute mark were located very close to one another. Based on the chromatogram obtained from the separation of the extract (Fig 14), the peak of interest was identified as the 14th peak produced at 9.189 minutes, due to its congruence with the cyanidin chloride standard. The identity of the peak was confirmed by spiking the samples with the standard solution due to the low height and peak area of the target compound in the chromatogram.

The extract contained 3.491±0.106 mg g^-1^ cyanidin (mean ± standard deviation), based on the calibration curve generated for the cyanidin chloride standard. Results from both visualisation and HPLC analyses suggest that the red abaxial layers of *L*. *discolor* are influenced by a concert of anthocyanins, rather than cyanidin alone.

## Discussion

Leaf morphology influences how light energy is utilised and maximised during photosynthesis [21]. Monocotyledonous leaves may present with either unifacial configuration, as observed in irises, or dorsiventral arrangement, similar to that observed in dicotyledonous leaves, for instance in *Musa* [38]. Clearly dorsiventral leaves are uncommon in the monocotyledonous group of plants. In this study, the mesophyll layers of *Anoectochilus* sp. and *L*. *discolor* were dorsiventral or heterogeneous in nature and were clearly defined. A single palisade mesophyll layer, rich in chloroplasts, was observed on the adaxial side of the leaves of both orchid species. Anthocyanin localisation was observed to specifically occur in the spongy layer. Similar characteristics were found in the water poppy family (Limnocharitaceae), differing only in the presence of large air cavities in the spongy mesophyll layer [38] when compared to the Jewel orchids in this study.

A plant’s photosynthetic response to shade conditions may occur in the form of changes and conformation in the plant structure, and biochemical responses in the chloroplasts [22]. The top epidermal layer of cells in the leaves of both the Jewel orchids in this study was composed of triangular-shaped cells that were devoid of organelles. It is known that plants in the forest understory may possess leaves with epidermal cells that help to focus light into the mesophyll layer to boost cellular illumination intensities [23, 39]. The light intensity illuminating the mesophyll layer of the leaf is amplified through the presence of epidermal or sub-epidermal cells that function as light-focusing lenses [22]. It is also worth noting that the chloroplasts found in the palisade mesophyll layers of *Anoectochilus* sp. and *L*. *discolor* displayed rearrangements and movements, indicating response to light levels. Chloroplast photorelocation movement promotes photosynthesis through utilisation of light signals in order to protect plants from high irradiation stress [40–45]. Although this aspect was not studied deeply in this research, light-dependent chloroplast movements are often observed in aquatic plants, for example *Vallisneria gigantea*, and in understorey shade species such as *Oxalis oregano* [22]. Leaf absorptance, known as the effectiveness of a leaf in absorbing radiant energy, is dependent on the location of the chloroplasts and the chlorophyll concentration in the leaf, as well as the path length of the light traversing the leaf [22]. Intense light irradiation causes the relocation of chloroplasts along the vertical walls of a cell and parallel to the light rays, while low-intensity irradiations induce chloroplasts to position themselves along the lower cell walls and perpendicular to the light rays [22, 46].

In this study, a strong red autofluorescence was obtained naturally when freehand sections of the leaves of both Jewel orchid species were viewed under UV, blue, and especially under green light illuminations. Anthocyanins are strongly indicated as the group of compounds that naturally produces red fluorescence in many plant species. Anthocyanins and azulenes, found in some plant cells, emit red fluorescence at maximum wavelengths of between 600–630 nm [47]. All major anthocyanins of *Arabidopsis* fluoresced red [48, 49]. Anthocyanins are known to be present in a localised or generalised manner in a number of plant species, usually in concert with an abaxial leaf layer which is dark green in colour [50]. Leaves of *Tipularia* and *Psilochilus*, for example, are known to possess green adaxial and purple abaxial layers. Most Jewel orchid plants possess leaves with a prominent abaxial anthocyanin layer that work in concert with chlorophyll in rendering the interveinal segments of the leaves a dark green, with chlorophyll-free regions stained red or purple [51]. Anthocyanin spots also occur on the leaves of *Cypripedium*, *Dactylorhiza*, *Orchis*, *Paphiopedilum*, *Phaius* and *Psychopsis* [50]. The anthocyanins present in the abaxial layers of leaves of *Goodyera* were postulated to assist in harvest of energy under low light conditions [31, 52]. However, details surrounding such orchids, especially in the case of Asian species, remain speculative [31].

It is known that both low and high light may impose stress on plants. The former imposes a limit on photosynthesis, and hence affects plant growth and net carbon gain. High light illumination may harm the photosynthetic apparatus [22]. The presence of anthocyanins is said to offer a measure of protection against excess light in aging leaves of deciduous trees [53, 54], in the overwintering of mature leaves or evergreen plants [55], and in young, developing foliage [26, 56]. Since anthocyanins were observed in both young and old leaves of both *Anoectochilus* sp. and *L*. *discolor*, there is a possibility that the anthocyanins may serve to protect the photosynthetic system in juvenile leaves or plants from excessive PAR. Hughes *et al*. [57] discovered a significant difference in the absorption of green light between the green and red abaxial tissues of *Begonia heracleifolia*. The anthocyanic tissues were found to absorb significantly higher levels of blue-green light when compared to acyanic tissues. Chloroplasts located within the anthocyanic abaxial layer of the leaves were also observed to display lower levels of fluorescence at these wavelengths. Gould *et al*. [58] hypothesised that this adaptation is integral in attenuating high irradiance levels resulting from sunpatches or sunflecks in understorey plants [57]. Anthocyanins located in the abaxial layers of the leaves are said to protect the plant by absorbing irradiance high enough to penetrate the mesophyll while allowing light absorption at lower irradiance levels, contrary to the backscatter theory [57]. However, it is worth noting that anthocyanic cells occurred in multiple layers in both the orchid species in this study, with a prominent anthocyanic layer occurring just beneath the palisade mesophyll of *Anoectochilus* sp., contrary to the study above. This raises the possibility that the anthocyanins present in *Anoectochilus* sp. and *L*. *discolor* may indeed have a role in mitigating damages that result from sudden high light conditions while serving to boost illumination to the chloroplasts under shady conditions.

The cyanidin chloride standard employed in this study was observed to produce two absorption peaks, at circa 280 and 520 nm, when scanned under UV and visible light wavelengths using the spectrophotometer. The same results were obtained when the standard was applied in the HPLC. Attempts were made to scan the methanolic extracts at 520 nm, the wavelength typically used in detecting anthocyanins, but no peaks corresponding to that of the standard were obtained at the desired retention period. The target peak could only be identified when the sample was scanned at 280 nm. Compounds that can be detected at 280 nm include an array of phenolic compounds such as catechins, flavan-3-ols, flavanone glycosides, benzoic acid derivatives, hydroxycinnamic acids, derivatives of oleuropein, ferulic acid dehydrodimers, procyanidins and anthocyanins [59]. Procyanidin is composed of the phenolic compounds (+)-catechin and (−)-epicatechin [60]. The results implied that the methanolic extracts of *L*. *discolor* may be composed of procyanidin, a proanthocyanidin that has not been degraded to cyanidin, hence its detection at solely 280 nm. However, the molar absorption coefficient of procyanidins was observed to be low when compared to other phenolic acids and flavonoids, during detection at 280 nm [61]. This was also observed in this study as the peak corresponding to the presence of cyanidin was relatively low when compared to the height of the other peaks, which may have corresponded to the presence of other phenolics. Furthermore, polyphenols such as chlorogenic acid, gallic acid, kaempferol and rutin were detected in *A*. *roxburghii*, hinting at the presence of such compounds within the orchid genus and possibly tribe [62]. A number of orchids contain cyanidin-based compounds. Flowers of orchid hybrids derived from the genus *Aranda*, *Renanthera* and *Vanda* are known to contain only cyanidin. Cyanidin-based compounds were also found in other orchid species: *Cymbidium finlaysonianum*, *Grammatophyllum speciosum* and *Pogonia japonica* were found to contain cyanidin-3-glycoside, while *Dendrobium* Caesar was found to contain cyanidin-3-rutinoside [30, 31].

## Conclusion

The Jewel orchids *Anoectochilus* sp. and *L*. *discolor* presented with typical morphologies of a monocotyledonous plant, with the exception of the leaves. Both the Jewel orchids possessed leaf with reticulate venation and mesophyll layers that were dorsiventral or heterogeneous in nature. The palisade mesophyll layer was rich in chloroplasts, while the spongy layer possessed lesser number of minute-sized chloroplasts. Anthocyanin localisation was observed to specifically occur in the spongy layer. Cyanidin was detected in the methanolic leaf extracts of *L*. *discolor*. The observations indicated that the leaves of *Anoectochilus* sp. and *L*. *discolor* may be adapted to maximise light harvest under low light conditions while rendering protection from sudden bursts of high irradiation.

## Supporting information

S1 Appendix Data used in the generation of the calibration curve of the cyanidin standard. The curve (A) was used to calculate the amount of cyanidin present in the powdered leaf sample of *L*. *discolor* (B).
(XLSX)
